# Overdose of Chloral Hydrate

**Published:** 1871-06

**Authors:** 


Overdoses of Chloral Hydrate.—The *Medical Times and Gazette* mentions several cases which tend to show that chloral hydrate may prove fatal when administered in too large a dose. One, taken from the *New York Journal of Psychological Medicine*, was that of a lady, exceedingly nervous, who had been subjected unavailingly to a great variety of treatment. At last chloral hydrate was given in six cumulate doses of thirty grains each. The sleep so induced, although every effort was made to arouse her, ended in death. The cerebral vessels were enormously congested. The patient had previously been taking bromide of potassium. In one of our metropolitan hospitals a fatal issue has followed the administration of a large dose of chloral; but here the patient was in an exhausted state from a severe operation. In Philadelphia a woman swallowed an enormous quantity of the drug (460 grains, it is believed). The symptoms were very severe; but remedies being applied promptly, she recovered.

